# Effect of the angiotensin receptor blocker irbesartan on metabolic parameters in clinical practice: the DO-IT prospective observational study

**DOI:** 10.1186/1475-2840-6-36

**Published:** 2007-11-27

**Authors:** Klaus G Parhofer, Felix Münzel, Michael Krekler

**Affiliations:** 1 Medical Department II – Grosshadern, University Munich, Marchioninistr. 15, 81377 Munich, Germany; 2 Bristol-Myers Squibb GmbH, Sapporobogen 6-8, 80637 Munich, Germany

## Abstract

**Aims:**

A number of intervention studies have shown that therapy with angiotensin receptor blockers, such as irbesartan, can improve metabolic parameters and reduce the incidence of diabetes mellitus. It is unknown whether this observation also holds true in routine clinical settings.

**Methods:**

We evaluated the effect of irbesartan (150 mg or 300 mg/d) together with or without hydrochlorothiazide (12.5 mg/d) in 3259 German patients. A total of 750 primary care physicians evaluated up to 5 subsequent patients with metabolic syndrome (58.9% diabetic), in whom irbesartan therapy was newly initiated (87%) or continued (13%).

**Results:**

Six months of irbesartan therapy decreased systolic blood pressure by 14% (157.4 ± 14.7 vs. 135.0 ± 10.7 mmHg) and diastolic blood pressure by 13% (92.9 ± 9.2 vs. 80.8 ± 6.8 mmHg). This was associated with a decrease in body weight (-2.3%), fasting glucose (-9.5%), HbA1c (-4.6%), LDL-cholesterol (-11%), triglycerides (-16%) and gamma-GT (-12%) and an increase in HDL-cholesterol (+5%). These changes were somewhat more pronounced in male than in female patients and in obese than in lean patients. Changes in glucose concentration and HbA1c were much more prominent in diabetic patients.

**Conclusion:**

Irbesartan therapy improves metabolic parameters in routine clinical settings. Thus, our study confirms previously published results from large intervention trials and extends the findings to routine clinical practice.

## Introduction

The metabolic syndrome which describes the concomitant presence of visceral obesity, insulin resistance or glucose intolerance, atherogenic dyslipidemia and hypertension is highly prevalent (approximately 25%) in Germany. The risk of developing diabetes mellitus type 2 for subjects with the metabolic syndrome is increased five-to nine-fold [1,2]. In addition, all components of the metabolic syndrome are common cardiovascular risk factors. Therefore patients with this syndrome have a two-four fold increase in risk for cardiovascular disease and death [3].

Large clinical trials evaluated the effect of angiotensin receptor blockers (ARB) on cardiovascular end points, generally showing very positive results. Therefore ARB and ACE-Inhibitors are now considered first line therapy in hypertensive patients with the metabolic syndrome [4,5]. An analysis of co-morbidity showed that such therapy also substantially lowers the risk for type 2 diabetes compared with other antihypertensive drugs and compared with placebo [6-8]. Furthermore, it lowers cardiovascular mortality independent of its antihypertensive effects. The reduced incidence of diabetes in ARB trials was associated with lowering of blood pressure, but independent of other predictors for diabetes development. Furthermore, an anti-inflammatory effect by treatment was observed, as well as an influence on the metabolism of free fatty acids, which could mediate the observed reduction in type 2 diabetes incidence. On the other hand, a recently published intervention trial which specifically aimed at reducing the incidence of type 2 diabetes with the ACE inhibitor ramipril failed to show a significant reduction in new onset diabetes, although average glucose concentration was decreased compared to placebo [9].

Thus, a large body of data indicates that interfering with the angiotensin system has positive effects on metabolic parameters. However, it is unknown whether these positive effects can also be observed in daily routine clinical practice, although recently published data from a post authorization trial indicate that this may be the case [10]. We therefore conducted an observational study in a large group of German hypertensive patients. The aim of this study was to evaluate the effect of irbesartan with and without hydrochlorothiazide on parameters of glucose metabolism, lipid metabolism, renal function, and blood pressure.

## Methods

A total of 3259 patients participated in this prospective, 6-month, open-label, observational, post-authorization survey. The patients were recruited from 649 general practitioners throughout Germany, who included up to 5 subsequent patients who fulfilled the inclusion criteria specified below and who at the time of the index visit were either already on irbesartan therapy or in whom irbesartan therapy was started at that time point. All subjects had elevated blood pressure (>130/85 mmHg or antihypertensive therapy). In addition, at least two of the following criteria were present: abdominal obesity (waist-circumference >102 cm (male) or >88 cm (female)), elevated triglycerides (>150 mg/dl), decreased HDL-cholesterol (male <40 mg/dl, female <50 mg/dl), elevated fasting glucose (>110 mg/dl). The characteristics of the subjects are shown in Table 1.

**Table 1:** Parameters of study participants at baseline

**Parameter**	**All**	**Men**	**Women**
n	3259	64.1%	35.9%
Age [years]	61.5 ± 10.5	60.2 ± 10.0	63.7 ± 10.8*
BMI [kg/m^2^]	30.9 ± 4.8	30.8 ± 4.4	31.0 ± 5.3
Waist-circumference [cm]	106.4 ± 13.6	110.2 ± 11.9	99.2 ± 13.7*
RR_systolic _[mmHg]	157.4 ± 14.7	157.5 ± 14.5	157.2 ± 15.1
RR_diastolic _[mmHg]	92.9 ± 9	93.3 ± 9.1	92.2 ± 9.5*
Fasting plasma glucose (FPG) [mg/dl]	126.4 ± 37.1	126.6 ± 37.3	126.0 ± 36.7
LDL-cholesterol [mg/dl]	144.9 ± 38.2	144.8 ± 38.5	145.2 ± 37.5
HDL-cholesterol [mg/dl]	48.4 ± 31.8	46.2 ± 30.9	52.6 ± 33.2*
Triglycerides [mg/dl]	217.4 ± 114.9	221.1 ± 127.7	209.9 ± 85.8*
Hs-CRP [mg/l]	4.81 ± 7.95	5.13 ± 8.38	3.75 ± 6.26
Gamma-GT [U/l]	52.9 ± 47.1	58.7 ± 50.6	40.6 ± 34.8*

BMI = body-mass index; * p < 0.01 for intergender differences;

Each subject was evaluated three times: At baseline (index visit), after three months and after six months. Patients were evaluated using a standardized form with which data on duration of hypertension, duration of metabolic syndrome, nicotine and alcohol consumption, blood pressure, pulse, weight, height, waist circumference, medications, lab-values (HbA1c, fasting glucose, fasting LDL-cholesterol, fasting HDL-cholesterol, fasting triglycerides, creatinine, hs-CRP, gamma-GT) were collected.

Mean and standard deviation as well as median and range were used to describe the population. t-tests were used to evaluate intergender differences, differences between diabetic and non-diabetic patients, and differences between different doses. Non-parametric tests were used for statistical evaluation were appropriate. SAS, version 9.1 was used for statistical analysis. The analysis was performed by GKM – Gesellschaft fuer Therapieforschung, Munich, Germany.

## Results

In this cohort of German hypertensive patients 49.1% did not take any antihypertensive medications at the index visit, while 50.9% were taking at least one antihypertensive drug. At baseline 423 patients (13.2%) were taking irbesartan, while in all others irbesartan therapy was initiated at the index visit. Furthermore, the patients were also taking a significant number of other medications (Table 2). This concomitant medication did not change during the observation period.

**Table 2:** Accompanying diagnosis and medication at baseline

**Parameter**	**n**	%*
Diabetes mellitus	1921	58.9
Hepatic steatosis	1258	38.6
Coronary artery disease	773	23.7
Benign prostatic hyperplasia	365	11.2
Nephropathy	315	9.7
Cardiac failure	306	9.4
Myocardial infarction	272	8.3
Cerebrovascular event	176	5.4
Active smoker	680	20.9
Former smoker	1012	31.1
Statins	1471	45.1
Orale antidiabetic drugs	1298	39.8
Betablockers	1088	33.4
Platelet Inhibitors	957	29.4
Calciumantagonists	735	22.6
Diuretics	637	19.5
Insulin	420	12.9
ACE-Inhibitors	337	10.3
Fibrates	200	6.1

Medications reported in less than 3% are not indicated in the table; * sum >100% because some patients were taking more than 1 medication.

In this study 594 patients (18.8%) were treated with irbesartan 150 mg/d, 665 patients (21%) with irbesartan 300 mg/d, 754 patients (23.8%) with 150 mg irbesartan and 12.5 mg hydrochlorothiazide and the rest (36.4%) with irbesartan 300 mg/d and 12.5 mg hydrochlorothiazide. During the observation period the number of patients treated with irbesartan 150 mg/d decreased slightly (from 42.6% to 37.0%) while those groups taking 300 mg/d increased (from 57.4% to 63%).

Within the observation period (6 months) systolic blood pressure decreased from 157.4 ± 14.7 mmHg to 135.0 ± 10.9 mmHg (-22.3%). Diastolic blood pressure decreased from 92.9 ± 9.2 mmHg to 80.8 ± 6.8 mmHg (-12.1%). Thus, the rate of patients with systolic blood pressure <130 mmHg increased from 1.6% to 20.4%. While the rate of patients with systolic blood pressure >180 mmHg decreased from 9.4 to 0.4%. Similarly, diastolic blood pressure was <85 mmHg in 15.7% at the index visit and 67% after 6 months of therapy. The rate of patients who had blood pressure <140/90 mmHg increased from 4.9 to 59.2%.

During the observation period the body weight decreased from 91.4 ± 15 kg to 89.3 ± 14.6 kg (-2.2%). This decrease was somewhat more pronounced in men (-2.5%) than in women (-1.4%). Similarly waist circumference decreased from 106.4 to 104.3 cm (all: -2.1%; men -2.6%; women -1.3%).

The effect on metabolic parameters is shown in Table 3. Overall we observed a reduction in HbA1c, fasting glucose, LDL-cholesterol, fasting triglycerides, Hs-CRP, Gamma-GT and an increase in HDL-cholesterol. Generally, these changes in metabolic parameters were more pronounced in patients receiving 300 mg irbesartan/d compared to those receiving 150 mg/d (Table 4). The effect on metabolic parameters was independent of the medication that the patients were receiving at the index visit.

**Table 3:** Effect of irbesartan on metabolic parameters in patients with metabolic syndrome

**Parameter**	**Baseline**	**On Therapy**	**Change^a^**	**Change^b^**	**p-value^c^**
Body weight [kg]	91.4 ± 15.0	89.3 ± 14.6	-2.1 ± 7.9	-2.3	< 0.0001
Waist-circumference [cm]	106.4 ± 13.6	104.3 ± 13.2	-2.1 ± 7.9	-2.0	< 0.0001
RR_systolic _[mmHg]	157.4 ± 14.7	135.0 ± 10.7	-22.3 ± 15.3	-14.2	< 0.0001
RR_diastolic _[mmHg]	92.9 ± 9.2	80.8 ± 6.8	-12.1 ± 9.9	-13.0	< 0.0001
Glucose [mg/dl]	126.4 ± 37.1	114.5 ± 29.4	-12.0 ± 30.7	-9.5	< 0.0001
HbA1c [%]	6.74 ± 1.14	6.43 ± 0.91	-0.31 ± 0.75	-4.6	< 0.0001
LDL-cholesterol [mg/dl]	144.9 ± 38.2	129.1 ± 31.8	-15.8 ± 30.5	-10.9	< 0.0001
HDL-cholesterol [mg/dl]	48.4 ± 31.8	51.0 ± 15.9	2.6 ± 31.8	5.4	< 0.0001
Triglycerides [mg/dl]	217.4 ± 114.9	182.5 ± 76.7	-34.9 ± 100.9	-16.1	< 0.0001
Gamma-GT [U/l]	52.9 ± 47.1	46.8 ± 38.9	-6.1 ± 30.2	-11.5	< 0.0001
Hs-CRP [mg/l]	4.81 ± 7.95	4.01 ± 6.74	-0.79 ± 6.72	-16.4	< 0.0001

^a ^absolute change; ^b ^relative change (%); ^c ^p-values for absolute change

**Table 4:** Dose dependent effects of irbesartan on metabolic parameters in patients with metabolic syndrome

**Parameter**	**150 mg/d^a^**	**300 mg/d^a^**	**p-value^b^**	**150 mg/d + HCT^a^**	**300 mg/d + HCT^a^**	**p-value^c^**
Glucose [mg/dl]	-9.1 ± 30.5	-12.3 ± 34.7	0.092	-7.8 ± 23.9	-14.3 ± 30.7	< 0.0001
HbA1c [%]	-0.24 ± 0.8	-0.30 ± 0.8	0.223	-0.26 ± 0.6	-0.34 ± 0.7	0.035
LDL-cholesterol [mg/dl]	-11.2 ± 26.92	-16.6 ± 30.4	0.004	-12.4 ± 26.4	-17.3 ± 33.3	0.001
HDL-cholesterol [mg/dl]	1 ± 37.4	2.8 ± 25.6	0.393	2.1 ± 22.6	4.1 ± 33.6	0.156
Triglycerides [mg/dl]	-27.4 ± 75.8	-31.0 ± 73.6	0.440	-30.9 ± 68.9	-40.3 ± 133.9	0.060
Gamma-GT [U/l]	-4.7 ± 37.2	-7.3 ± 24.2	0.257	-4.2 ± 21.7	-6.4 ± 32.4	0.142
Hs-CRP [mg/l]	-0.42 ± 2.9	-0.80 ± 10.5	0.704	-0.47 ± 1.4	-1.25 ± 7.2	0.111

^a ^absolute change; ^b ^relates to difference between irbesartan 150 mg/d and irbesartan 300 mg/d; ^c ^relates to difference between irbesartan 150 mg/d with HCT (12.5 mg/d) and irbesartan 300 mg/d with HCT (12.5 mg/d).

**Figure 1:**
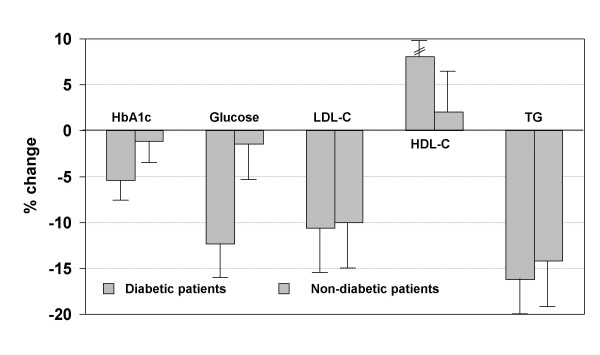
Effect of irbesartan on metabolic parameters in diabetic and non-diabetic patients with metabolic syndrome. All changes from baseline are significant (p < 0.001). Differences between diabetic and non-diabetic subjects are only significant for HbA1c (p < 0.0001), fasting glucose (p < 0.0001), and HDL-cholesterol (p = 0.05), while differences between diabetic and non-diabetic patients were not significant concerning triglycerides (p = 0.148) and LDL-cholesterol (p = 0.819).

All metabolic changes were more pronounced in men than in women, in younger (<62 years) than in older (≥62 years), in more obese (BMI ≥30 kg/m^2^) than in leaner patients. HbA1c and fasting glucose reduction was significant in diabetic and non-diabetic patients, but was much more pronounced in those with diabetes. All other parameters decreased in diabetic and non-diabetic patients, however, improvement was overall more pronounced in patients with diabetes (Figure 1). Within the group of diabetic patients we did not observe an influence of the underlying anti-diabetic treatment on the metabolic efficacy of irbesartan (data not shown). In a multivariate analysis it was shown that changes in HbA1c and glucose were mostly related to BMI while changes in LDL-cholesterol and triglycerides were related to age. Furthermore, triglycerides, HDL-cholesterol and HbA1c were correlated with sex.

All metabolic changes were significantly correlated with the changes in blood pressure, although the correlation coefficients were low (Table 5). Changes in systolic blood pressure correlated best with changes in HbA1c, while changes in diastolic blood pressure correlated best with changes in LDL-cholesterol and triglycerides.

**Table 5:** Correlations between changes in blood pressure and changes in metabolic parameters

**Parameter**	**Systolic blood pressure**		**Diastolic blood pressure**	
	**r**	**p**	**r**	**p**
HbA1c [%]	0.114	<0.0001	0.064	0.002
Glucose [mg/dl]	0.078	<0.0001	0.045	0.016
LDL-cholesterol [mg/dL]	0.097	<0.0001	0.132	<0.0001
HDL-cholesterol [mg/dL]	-0.025	ns	-0.064	0.001
Triglycerides [mg/dl]	0.085	<0.0001	0.115	<0.0001

## Discussion

In our study performed in routine clinical practice six months of irbesartan therapy resulted in a considerable improvement in a number of metabolic parameters. We observed a significant reduction in HbA1c, fasting glucose, LDL-cholesterol, fasting triglycerides, gamma-GT and hs-CRP.

These changes in metabolic parameters were somewhat dose dependent, thus more pronounced in patients receiving 300 mg irbesartan compared to those receiving 150 mg/d. Concomitant therapy with thiazide did not affect these changes. Overall the metabolic changes were more pronounced in men and more pronounced in overweight and obese subjects. The observed changes were strongly related to changes in blood pressure.

Our observations are consistent with previous intervention trials. Large clinical trials showed that ARB therapy substantially lowers the risk for type 2 diabetes compared to other antihypertensive therapies or placebo [6-8]. Furthermore, in a recently published subanalysis of the Treat to Target post authorization survey in 14200 patients very similar results were observed. There was a significant improvement in blood pressure and metabolic risk factors as a result of Irbesartan treatment. Similar to our study there was no evidence of a difference between monotherapy and combination therapy with hydrochlorothiazide with regard to the cardiovascular risk profile [10]. While Kintscher and colleagues [10] investigated hypertensive patients with and without metabolic syndrome, we studies only metabolic syndrome patients including a large number of diabetic patients. Despite this difference the over-all results are very similar indicating that a broad range of patients benefit from such therapy with respect to metabolic parameters. However, it should also be noted that in both studies patients with supposedly more severe insulin resistance benefited the most.

In addition, a number of small clinical trials have evaluated the effect of ARB therapy on individual components of the metabolic syndrome particularly on glucose metabolism [11-13]. It is currently unclear whether individual ARB differ in their potential to improve metabolic parameters, although telmisartan and irbesartan seem to be particularly powerful in this respect [14,15].

Several mechanisms could link ARB therapy with an improvement in metabolic parameters. ARB may improve insulin sensitivity by affecting inflammatory processes and altering free fatty acid concentration and/or metabolism [16]. Furthermore, ARB could directly stimulate PPAR-gamma [17-19].

Our study extends the known findings to the "every day" routine clinical practice. The magnitude of improvement is similar to that seen in placebo controlled intervention trials. It is however important to confirm these studies in more typical settings, because patients included in placebo controlled intervention trials are usually highly selected which could bias results. On the other hand our open-label, observational design also results in considerable limitations. Thus, it should be noted, that all subgroups including those that were already on ARB therapy or ACE inhibitor therapy at the beginning benefited with respect to their metabolic parameters. This indicates that part of the improvement in metabolic parameters is related to the fact that the patients were included in this observational study (study-in effect). Notably, we also observed a small but significant decrease in body weight, which also indicates that some of the beneficial effects observed during the study are related to the study-in effect. On the other hand, as outlined above, irbesartan stimulates PPAR-gamma dependent pathways and thus results in an altered flux of free fatty acids, which may affect nutritional behavior and caloric intake. Furthermore, metabolic improvement was related to blood pressure reduction. It therefore cannot be decided whether the improvement in metabolic parameters is specific for irbesartan (or ARB therapy) or the result of blood pressure reduction. However, a number of large intervention trials have shown that the effect of ARB therapy on metabolic parameters reaches beyond blood pressure reduction.

## Conclusion

The present study indicates that hypertensive patients with metabolic syndrome with and without diabetes benefit from irbesartan therapy not only with respect to blood pressure reduction but also with respect to a significant improvement of metabolic parameters such as glucose, lipids and liver values.

## Competing interests

KGP has received speaker's fees from Bristol-Myers Squibb.

## Authors' contributions

KG participated in the design of the study and the analysis of the data and drafted the manuscript. FM participated in the design of the study and helped to draft the manuscript. MK conceived of the study, and participated in its design and coordination and helped to draft the manuscript. All authors read and approved the final manuscript.
